# *In silico *and microarray-based genomic approaches to identifying potential vaccine candidates against *Leptospira interrogans*

**DOI:** 10.1186/1471-2164-7-293

**Published:** 2006-11-16

**Authors:** Hong-Liang Yang, Yong-Zhang Zhu, Jin-Hong Qin, Ping He, Xu-Cheng Jiang, Guo-Ping Zhao, Xiao-Kui Guo

**Affiliations:** 1 Department of Microbiology and Parasitology, Shanghai Jiao Tong University School of medicine. 200025 Shanghai, PR China; 2 Institute of Medical Biology, PeKing Union Medical College & Chinese Academy of Medical Sciences. 650118 Kunming, PR China; 3 Department of Pathology, Shanghai Jiao Tong University School of medicine. 200025 Shanghai, PR China; 4 Laboratory of Molecular Microbiology, Institute of Plant Physiology and Ecology, Shanghai Institutes for Biological Sciences, Chinese Academy of Sciences, 200032 Shanghai, PR China

## Abstract

**Background:**

Currently available vaccines against leptospirosis are of low efficacy, have an unacceptable side-effect profile, do not induce long-term protection, and provide no cross-protection against the different serovars of pathogenic leptospira. The current major focus in leptospirosis research is to discover conserved protective antigens that may elicit longer-term protection against a broad range of *Leptospira*. There is a need to screen vaccine candidate genes in the genome of *Leptospira interrogans*.

**Results:**

Bioinformatics, comparative genomic hybridization (CGH) analysis and transcriptional analysis were used to identify vaccine candidates in the genome of *L. interrogans *serovar Lai strain #56601. Of a total of 4727 open reading frames (ORFs), 616 genes were predicted to encode surface-exposed proteins by P-CLASSIFIER combined with signal peptide prediction, α-helix transmembrane topology prediction, integral β-barrel outer membrane protein and lipoprotein prediction, as well as by retaining the genes shared by the two sequenced *L. interrogans *genomes and by subtracting genes with human homologues. A DNA microarray of *L. interrogans *strain #56601 was constructed for CGH analysis and transcriptome analysis *in vitro*. Three hundred and seven differential genes were identified in ten pathogenic serovars by CGH; 1427 genes had high transcriptional levels (Cy3 signal ≥ 342 and Cy5 signal ≥ 363.5, respectively). There were 565 genes in the intersection between the set encoding surface-exposed proteins and the set of 307 differential genes. The number of genes in the intersection between this set of 565 and the set of 1427 highly transcriptionally active genes was 226. These 226 genes were thus identified as putative vaccine candidates. The proteins encoded by these genes are not only potentially surface-exposed in the bacterium, but also conserved in two sequenced *L. interrogans*. Moreover, these genes are conserved among ten epidemic serovars in China and have high transcriptional levels *in vitro*.

**Conclusion:**

Of the 4727 ORFs in the genome of *L. interrogans*, 226 genes were identified as vaccine candidates by bioinformatics, CGH and transcriptional analysis on the basis of the theory of reverse vaccinology. The proteins encoded by these genes might be useful as vaccine candidates as well as for diagnosis of leptospirosis.

## Background

Leptospirosis is a globally important zoonotic disease caused by pathogenic *Leptospira *species[1]. Leptospires are thin, helically coiled, motile bacteria, classified into 17 genomospecies (including the saprophyte *Leptospira biflexa *and the pathogen *Leptospira interrogans*) on the basis of DNA-DNA hybridization studies, or serologically classified into more than two hundred pathogenic serovars on the basis of structural heterogeneity in the carbohydrate component of the lipopolysaccharide[2,3]. Currently available vaccines, based on inactivated whole bacteria or membrane preparations from pathogenic leptospires, are of low efficacy, have an unacceptable side-effect profile, require annual booster immunizations and do not confer cross-protective immunity against different serovars [4-6]. Because of these concerns, the current major focus in leptospirosis research is to discover cross-species-conserved or cross-serovar-conserved protective antigens that may elicit longer-term protection against a broad range of *Leptospira*[5,7]. New vaccine development strategies are thus needed for preventing this zoonosis. Reverse vaccinology, which based on the genomic approach, has been applied to some bacteria, and novel vaccine candidate sequences have been identified [8-11]. The genome projects of two *Leptospira *strains give us intensive knowledge on the whole genome level [12-14]. Although many efforts have been made to identify the surface-exposed proteins of leptospires, finding perfect vaccine candidate antigens that provide cross-protection against different serovars of pathogenic *L. interrogans *still requires further work[7,15-17].

In our current study, we identified 226 potential candidate vaccine genes against *L. interrogans *using *in silico *analysis, comparative genomic hybridization (CGH) and transcriptional analysis, based on a genome-wide DNA microarray comprising 3528 open reading frames (ORFs) derived from the original annotation of *L. interrogans *strain #56601. These candidate genes not only encode surface-exposed proteins of *L. interrogans *strain #56601, but also have high transcription levels *in vitro*. Moreover, the proteins encoded by these genes are conserved in two sequenced *L. interrogans *and ten epidemic pathogenic serovars in China.

## Results

### *In silico *analysis for identification of genes encoding surface-exposed proteins

In 4727 ORFs of *L. interrogans *strain #56601, 1282 proteins were predicted to be surface-exposed using P-CLASSIFIER, 654 proteins had signal peptides, 813 were predicted to have no more than four α-helices with transmembrane topology, 96 were predicted to have β-barrel topology implying that they are integral β-barrel outer membrane proteins, and 158 were predicted have a lipoprotein signal peptide using SpLiP. The number of genes in the intersection between the set of surface-exposed proteins identified by P-CLASSIFIER and the set of proteins characterized by at least one of the four characteristic topologies is 688. We calculated the similarity of proteins between serovar Lai and serovar Copenhageni as well as between serovar Lai and human (cut-off value: similarity >70% and E value = 1e-10 for two serovars, E value = 1e-10 for serovar Lai and human) using BLASTP. We found 3672 orthologs between the two serovars, and 605 proteins that are similar in serovar Lai and human. Finally, 616 genes were yielded by the bioinformatics study by retaining the orthologs between the two serovars and subtracting the genes that were similar in serovar Lai and human.

### Comparative genomic hybridization

We prepared a gene chip microarray corresponding to the complete genome sequence of *L. interrogans *strain #56601. The chips were hybridized to labelled total DNA extracted from strain Fiocruz L1–130 and ten pathogenic serovars. On the basis of test hybridizations of strain Fiocruz L1–130 vs. the reference sample, we considered genes that gave hybridization ratios between 1.0 and 3.0 to be present in both strains and greater than 10.0 to be absent from the test strain. Ambiguous values between 3.0 and 10.0 may have been due to highly divergent genes or hybridization to paralogous genes. The CGH results revealed that 307 genes of *L. interrogans *strain #56601 were absent or highly divergent in at least one strain tested. After subtracting these 307 differential genes, we were left with 565 genes, which not only encode presumably surface-exposed proteins but also are conserved in the ten pathogenic serovars.

### Transcriptome analysis

**Figure 1:**
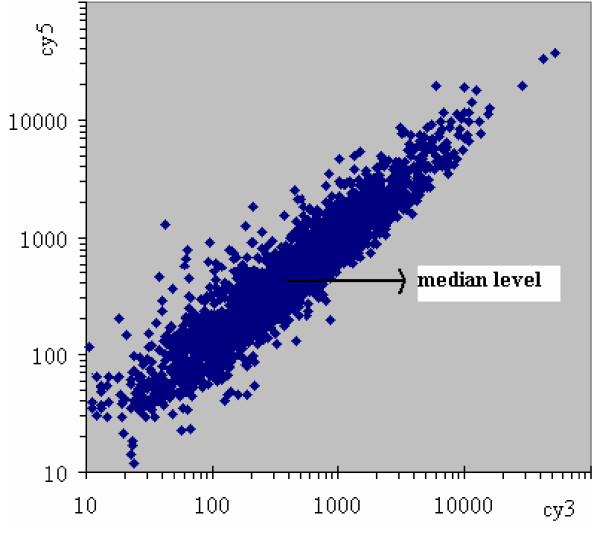
Identification of highly expressed genes in *L. interrogans *by microarray. Bacteria were grown in EMJH medium at 37°C and were collected when the culture reached mid-exponential-phase. RNA was purified and labelled with either Cy3 or Cy5 and hybridized with the microarray of *L. interrogans *strain #56601 (3528 genes). Transcription analysis revealed that 1427 genes were highly expressed (cy3 signal ≥ 342 and cy5 signal ≥ 363.5).

Microarray analysis of the mRNA extracted from *in vitro *grown leptospires revealed that the fluorescence signals of Cy3 and Cy5 ranged from 10.5 to 51,707 (see Figure 1); 1427 genes were expressed above the median level (Cy3 signal ≥ 342 and Cy5 signal ≥ 363.5) in the microarray and therefore as genes with high transcriptional levels. The intersection between the sets of 565 and 1427 genes contained 226 genes. Among them, 8.0% (18/226) were located extracellularly, 53.1% (120/226) in the outer membrane, 16.4% (37/226) in the periplasmic space and 22.6% (51/226) in the inner membrane according to predictions. These vaccine candidates were classified further according their gene names and clusters of orthologous groups (COGs) [18,19](Table 1, 2, 3, 4); 60.6% (137/226) of the candidates had COG annotations.

## Discussion

Vaccines composed of whole cells or outer membrane envelope are available in some countries to prevent human leptospirosis, and clinical trials have been reported [20-23]. In view of their disadvantages, especially their inability to elicit longer-term protection against different serovars of pathogenic leptospires, efforts have been focused on developing subunit vaccines[24]. During recent years, Hap1[25] (also known as LipL32[26]), LipL41, OmpL1[27] and Lig[28,29] proteins have been identified as promising vaccine candidates for preclinical trials.

The availability of complete genome sequence information for many pathogens and the development of sophisticated computer programs have led to a new paradigm in vaccine development. Now it is possible to screen potential vaccine candidate genes in a reverse manner starting from the genome. This reverse vaccinology was first applied to MenB[30] and is now applied routinely in vaccine development, as in the search for vaccines against *S. pneumoniae, Streptococcus agalactiae, Staphylococcus aureus, Porphyromonas gingivalis, Chlamydia pneumoniae *and other microorganisms[10]. Bioinformatics analysis is the first important strategy of reverse vaccinology. Gram-negative bacteria have five subcellular location sites: cytoplasm, inner membrane, outer membrane, periplasm and extracellular space. The surface-exposed proteins, i.e. those located in sites other than the cytoplasm, are the most suitable vaccine candidates because they are more susceptible to antibody recognition and can therefore elicit protective immune responses. Many sophisticated computer programs have been developed to predict the subcellular locations of putative proteins in the whole genome [31-33]. Analyzing the gene transcription profile using DNA microarrays provides a second vaccine candidate selection strategy in reverse vaccinology. A gene having a fluorescent signal above the median value corresponds to an expression level higher than 5–10 mRNA copies per genome[34]. Those highly expressed genes could be potential vaccine candidates[34]. Finally, other approaches such as proteomic technology can be used to screen vaccine candidates. Using combined these strategies, genes encoding potential vaccine antigens can eventually be identified.

In our preliminary selection, all genes in *L. interrogans *strain #56601 were searched using P-CLASSIFIER, a system for predicting the subcellular locations of proteins on the basis of amino acid subalphabets and a combination of multiple support vector machines[33]. Moreover, four topologies were predicted by the corresponding programs. Proteins predicted to be surface-exposed and having any of these four topologies were screened as preliminary vaccine candidates. All proteins with more than four predicted transmembrane spanning regions were removed from the list of candidates, not only because they are likely to be completely embedded in the cell membrane and therefore inaccessible to antibodies, but also because they are difficult to express in *E. coli*[34]. We retained the genes shared by the two sequenced serovars and subtracted genes that had human homologues. The reason we subtracted human homologues is they are likely to cause problems of autoimmunity[35]. Finally, we narrowed the list of vaccine candidates to 616 genes in the genome of *L. interrogans *strain #56601.

In order to explore vaccine candidates that could generate cross-protection against the diverse serovars of leptospires, we applied CGH to identify genes that are conserved among the ten pathogenic strains involved in most infections[36]. This approach allowed us to refine the vaccine candidate shortlist further by eliminating antigens that were not conserved among these serovars. The 565 vaccine candidates not only presumably surface-exposed but also conserved among the ten prevalent serovars in China were identified as the result of this approach.

Transcriptome analysis was performed using DNA microarrays of *L. interrogans *in order to assess the transcription levels of all genes in the genome. A graph of the signal obtained for each gene gave a diagonal distribution reflecting the expression level of that gene. After subtracting genes with transcriptional levels below the median, we were left with 226 genes as vaccine candidates.

Applying the theory of reverse vaccinology, 226 genes had been identified as potential vaccine candidates against *L. interrogans *combined bioinformatics, CGH and transcriptional analysis. Among them, 60.6% (137/226) have COG annotations; thus, nearly 40% either have an unknown function or have no COG annotation. This group of gene products offers great promise as it comprises a pool of previously unexploited vaccine targets. To evaluate our results, we compared our candidates with those identified by others. Gamberini et al. (2005) found approximately 20% potential surface proteins using *in silico *approach, and sixteen proteins were recognized by antibodies present in human sera[15]. However, only three of them (LA0222, LA2637 and LA2741) appear in our final set. This is not unexpected, since 206 genes encoding hypothetical or unknown proteins were selected from approximately 20% of the genome for cloning and expression. Nally et al. (2005) characterized 32 proteins in outer membrane vesicles of *L. interrogans *serovar Copenhageni by two-dimensional gel electrophoresis, including previously-described outer membrane proteins (OMPs); in addition, unknown, hypothetical and putative OMPs were also identified[17]. Interestingly, only two proteins (LA0222 and LA2637) are represented among the sixteen proteins found by Gamberini and co-workers. There is an overlap of eight genes between our result and that of Nally et al. (2005) (LA0222, LA0505, LA0616, LA1495, LA2024, LA2295, LA2637 and LA3091). The reasons responsible for the discrepancies among the results may be due to differing methodologies. Genomics, transcriptional profiling and proteomics have emerged in the post genomic era with potential to speed up the vaccine discovery research process. It should be pointed out that those methods have their respective advantages and limitations, and can be complementally utilized in the development of the novel vaccines. Genomics involves the use of various softwares to predict sublocalization of proteins. However, some algorithms have limited accuracy. Although transcriptome analysis uses gene chip array to measure gene expression but suffers from the fact that mRNA levels may not reflect protein levels. Expression of a transcribed gene may be regulated at the level of translation. It is believed that the proteome maps of microorganisms are important to understand cellular status at the protein level, which cannot be deciphered from genome or transcriptome analysis[37]. Proteomics of outer membrane can rapidly identify almost all proteins in outer membrane. However, some of the proteins identified in membrane preparations are in fact typical cytoplasmic proteins[10,38]. Moreover, one of the major disadvantages of subproteomic studies by 2-D gel electrophoresis and mass spectrometry is the potential for contamination via leaky fractionation or lysis[39]. Nally et al. (2005) also revealed that outer membrane vesicles contain small amounts of inner membrane or cytoplasmic proteins in their proteomic study[17]. It is worth mentioning here that mainly surface-exposed proteins such as LipL32 (LA2637)[26,40], LipL41 (LA0616)[27,40], LipL45 (LA2295)[41] and LipL21 (LA0011)[42] have higher transcriptional levels in our results; this suggests that the genes with higher transcriptional levels identified in our current research may be preferable for development as vaccine candidates.

This is the first time that CGH and transcription analysis have been used to identify potential candidates for vaccines against *L. interrogans*. Our present work corroborates previous studies, showing the advantages of reverse vaccinology[8,11]. The next step following our present research is to verify whether the selected vaccine candidates are surface-exposed and to evaluate the protective activities of these proteins. Such studies will lead to the development of safe and effective new vaccines against leptospirosis in the future.

## Conclusion

We have performed high-throughput *in silico *and microarray-based processes that are useful for determining potential vaccine candidates against leptospirosis. In total, 226 genes were identified in the genome of *L. interrogans *serovar Lai type strain #56601 using bioinformatics, CGH and transcriptional analysis. The proteins encoded by these genes are not only potentially surface-exposed in the bacterium, but also conserved in two sequenced *L. interrogans*. Moreover, these genes are conserved among ten epidemic serovars in China and have high transcriptional levels *in vitro*. These proteins might therefore be useful for vaccine candidates as well as for the diagnosis of leptospirosis. Further research, including verification that these vaccine candidates are surface-exposed and evaluation their protective activities, will aid in the study of vaccines against leptospirosis in the future.

## Methods

### Bacteria strains and growth condition

Ten strains of *L. interrogans *were used in this study (Table 5). All the strains were obtained from the Institute for Infectious Disease Control and Prevention (IIDC), Beijing, China. Leptospires were maintained by serial passages in guinea pigs for preservation of virulence and were cultured in liquid Ellinghausen-McCullough-Johnson-Harris (EMJH) medium at 28°C or 37°C with shaking under aerobic conditions. Culture conditions were then developed to ensure that only mid-exponential-phase bacterial cultures at a mean density of 10^6^/ml were used in further experimentation. The cells were harvested by centrifugation at 10,000 *g *for 10 min at 4°C.

The *L. interrogans *serogroup Icterohaemorrhagiae serovar Lai type strain #56601 (strain Lai) was used to construct the DNA microarray. The genomic DNA of strain Fiocruz L1–130 was kindly provided by the Centro de Pesquisas Goncalo Moniz.

### *In silico *analysis

Genes and protein data for human and for the sequenced *L. interrogans *(serovar Lai and serovar Copenhageni) were downloaded from NCBI. P-CLASSIFIER[33] was applied to predict the subcellular locations of proteins in *L. interrogans *strain #56601. Signal peptide prediction was carried out using SignalP 3.0[43]. α-Helix transmembrane topology prediction was carried out using TMHMM[44]. BOMP was used to predict β-barrel outer membrane proteins[45]. Putative lipoproteins were predicted by SpLiP[46]. To identify proteins orthologous between serovar Lai and serovar Copenhageni as well as between serovar Lai and human, all predicted proteins were searched against each other locally using BLASTP[47].

### Comparative genomic hybridization

DNA microarrays of *L. interrogans *strain #56601 consisting of 3528 annotated ORFs longer than 250bp were prepared as previously described [48]. The genomic DNA of *L. interrogans *strain #56601 was used for reference in the double-fluorescence hybridization, and the genomic DNA of strain Fiocruz L1–130 was used as a control. A CGH microarray analysis of strain Lai and strain Fiocruz L1–130 was performed first. The qualified threshold determined in this control experiment was used to identify gene deletions in other strains. Reference or test DNA was fluorescently labelled through direct incorporation of Cy3-dCTP or Cy5-dCTP (Amersham Pharmacia Biotech) respectively by a randomly primed polymerization reaction. Unincorporated nucleotides and random primers were removed using QIAquick Nucleotide Removal columns (QIAGEN) according to the manufacturer's instructions.

Hybridizations were conducted in a hybridization chamber at 42°C overnight. Slides were washed at 55°C with 1 × SSC containing 0.2% SDS for 10 min and then at 55°C with 0.1 × SSC containing 0.2% SDS for 20 min and finally at room temperature with 0.1 × SSC for 3 min. Competitive hybridization was performed twice for each strain. In the first experiment, *L. interrogans *strain #56601 reference DNA and the sample DNA were labelled with Cy3 and Cy5, respectively. In the second hybridization, the dyes for labelling were interchanged.

Microarrays were scanned using a Chipreader laser scanner GenePix 4000B AXON (Axon Instruments, Union City, CA) according to the manufacturer's recommendations. Spot quantification, signal normalization and data visualization were performed using the programs GeneSpring 5.0.2 (Silicon Genetics) and Microsoft Excel.

### Transcriptome analysis

*L. interrogans *was grown in EMJH medium at 37°C under aerobic conditions for transcriptome analysis. Only mid-log-phase cultures at a mean density of 10^6^/ml in 100 ml were used in transcriptional experiments.

Total RNA was isolated from leptospires using Trizol reagent (Invitrogen) according to the manufacturer's protocol. Contaminating DNA was digested with RQ1 RNase-free DNase (Promega Corp.). The treated RNA was purified with a QIAGEN RNeasy Kit (QIAGEN).

RNA (10 μg) was labelled with Cy3 by reverse transcription using Superscript α (Invitrogen). Unincorporated dye was removed using a QIAquick Nucleotide Removal Kit (QIAGEN) as specified in the manufacturer's protocol. Samples were hybridized under cover slides to the microarray slides overnight at 42°C, and then washed as usual. The hybridization slides were processed by Tiffsplit (Agilent) and data were further analyzed using Genespring software 5.0.2 and normalized using mean values combined with Microsoft Excel software. Microarrays were used to assay relative RNA abundance. Flagged spots or SN<2 spots were excluded for intrachip and interchip reproducibility analysis. We calculated the coefficients of three spots in same chip for each gene to estimate intrachip reproducibility using Microsoft Excel. The signal values from the experiments represent average mRNA abundances. As in the CGH experiments, the dyes for labelling Cy3 and Cy5 were interchanged in the second hybridization.

**Figure 2:**
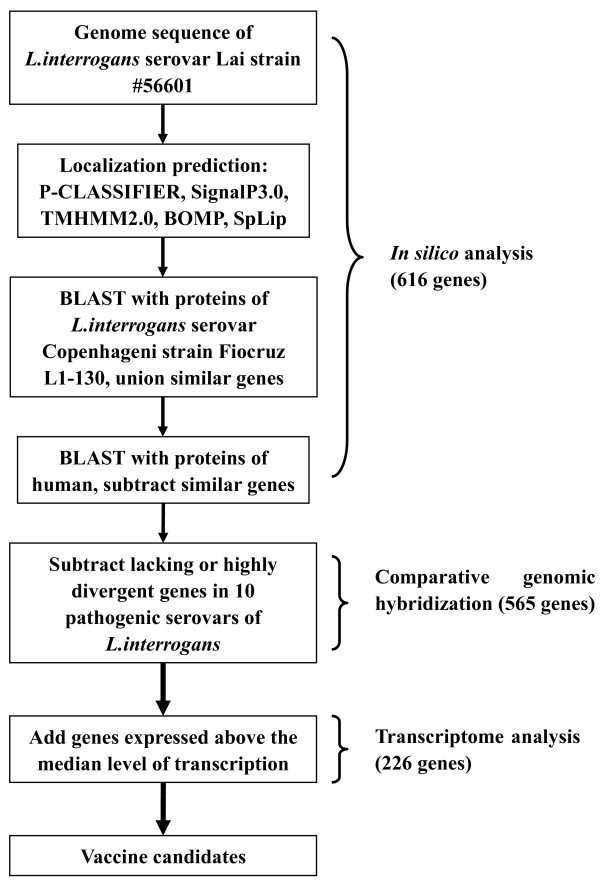
Schematic representation of general procedure to identify the vaccine candidates in the genome of *L. interrogans *(the numbers in parentheses are the results after the corresponding procedure step).

Figure 2 is a scheme of the procedure we used to identify the vaccine candidates as described above (the numbers in parentheses are the results after the corresponding procedure step).

## Authors' contributions

HLY and XKG designed the research project. HLY and YZZ carried out the bioinformatics analysis. PH and HLY completed the CGH. JHQ and HLY coordinated the transcriptome analysis. HLY and XKG drafted the manuscript. XCJ and GPZ participated in the design of the study and helped to draft the manuscript. All authors contributed to the writing and preparation of the manuscript. All authors read and approved the final manuscript.

## Figures and Tables

**Table 1:** The result of vaccine candidates according to localization sites: extracellular

gene	Cy3 signal	Cy5 signal	COG	product
LA0074	402.2	574.7	-	hypothetical protein
LA0322	1,118	760.5	-	hypothetical protein
LA0444	1,699	1,024	COG1196D COG4254S	hypothetical protein
LA0587	3,246	2,998	COG1075R	Lactonizing lipase
LA0617	672	487.5	-	hypothetical protein
LA1433	1,946	1,552	-	hypothetical protein
LA1508	559.2	853	-	putative outermembrane protein
LA1569	755.3	391.3	COG5651N	putative lipoprotein
LA2471	354.5	572.5	COG0457R	putative outermembrane protein
LA2823	1,466	884	-	putative lipoprotein
LA2975	478.8	454.2	-	hypothetical protein
LA2992	663.8	535.3	COG0419L	hypothetical protein
LA3210	410.7	847.7	-	hypothetical protein
LA3338	899.8	795.8	-	putative lipoprotein
LA3394	392.3	652	-	hypothetical protein
LA3779	374.8	431.7	-	hypothetical protein
LA3848	395.5	368.8	-	putative lipoprotein
LB225	798	1,069	-	hypothetical protein

**Table 2:** The result of vaccine candidates according to localization sites: outermembrane

gene	Cy3 signal	Cy5 signal	COG	product
LA0049	471	478.2	COG0840NT COG2202T	aerotaxis sensor receptor, flavoprotein
LA0099	1,081	853.5	-	hypothetical protein
LA0166	2,149	1,113	COG1196D	hypothetical protein
LA0178	1,224	953.8	COG0706U	60Kd inner membrane protein
LA0241	554.5	905.7	COG1999R	SCO1/SenC family protein
LA0253	755.8	559.2	COG2849S	hypothetical protein
LA0272	462.2	734.8	-	hypothetical protein
LA0301	787	771.8	COG2885M	outer membrane protein OmpA family
LA0330	554	397.3	COG2366R	Penicillin G acylase precursor
LA0339	1,060	1,185	COG0584C	Glycerophosphoryl diester phosphodiesterase
LA0365	1,290	1,959	-	hypothetical protein
LA0370	720.7	952.8	-	hypothetical protein
LA0378	692.5	560.3	COG0457R	TPR-repeat-containing proteins
LA0379	1,134	957	-	hypothetical protein
LA0410	3,451	3,604	COG2834M	hypothetical protein
LA0423	553	712.8	COG2931Q	hypothetical protein
LA0505	6,317	7,727	COG1409R	probable glycosyl hydrolase
LA0532	736.5	684.7	-	hypothetical protein
LA0568	434.8	435.3	COG2067I	hypothetical protein
LA0635	1,131	712.8	-	S-layer-like array protein
LA0678	973.3	952.5	COG0840NT	Methyl-accepting chemotaxis protein mcpB
LA0811	478.8	770.5	-	hypothetical protein
LA0818	481.3	946.8	-	hypothetical protein
LA0878	886.7	555.7	COG0266L	DshA protein
LA0940	1,467	1,026	-	hypothetical protein
LA0957	2,798	1,542	COG1538MU	outer membrane efflux protein
LA1009	764.8	887.7	COG5009M	Penicillin-binding protein 1A
LA1010	1,124	829	-	putative outermembrane protein
LA1087	376.5	536	-	hypothetical protein
LA1099	2,388	1,500	COG3103T	hypothetical protein
LA1100	2,901	2,796	COG1538MU	outer membrane efflux protein
LA1161	474.2	403	COG2067I	long-chain fatty acid transport protein
LA1174	615	423	COG0834ET	amino acid ABC transporter, periplasmic amino acid-binding protein
LA1192	616.3	545.3	-	putative outermembrane protein
LA1404	1,377	977	-	putative outermembrane protein
LA1412	1,034	640.3	-	hypothetical protein
LA1495	1,920	2,172	-	putative outermembrane protein
LA1501	558.2	545.7	COG4775M COG5009M	hypothetical protein
LA1507	1,615	1,747	-	hypothetical protein
LA1690	744.7	471.7	COG0449M	hypothetical protein
LA1733	1,418	1,557	-	hypothetical protein
LA1912	873.8	745	-	putative outermembrane protein
LA1917	595.5	535.3	-	hypothetical protein
LA1931	941.5	1,540	-	putative outermembrane protein
LA1987	909.3	994.5	-	putative outermembrane protein
LA1996	556.8	674	-	hypothetical protein
LA2024	2,594	2,079	-	hypothetical protein
LA2063	1,463	1,967	-	hypothetical protein
LA2094	548.2	380.3	COG1716T	FHA-domain containing protein
LA2126	1,223	979.7	COG0616OU	Putative signal peptide peptidase sppA
LA2215	1,045	679.2	COG1196D COG1360N	Chemotaxis motB protein
LA2238	420	464.7	COG0726G	polysaccharide deacetylase
LA2266	367.3	364.5	-	putative outermembrane protein
LA2267	886.2	1,542	COG0457R	putative outermembrane protein
LA2268	971.7	1,074	-	putative outermembrane protein
LA2295	4,445	6,689	COG0532J COG4254S	LipL45 protein
LA2318	813.2	673.8	COG4775M	Predicted outer membrane protein
LA2368	347.8	585	COG1555L COG3156U COG0477GEPR COG0075E	type II secretion pathway related protein etpK-like protein
LA2375	1,255	2,047	COG1450NU	General secretory pathway protein D
LA2377	377.5	418	COG0739M	peptidase, M23/M37 family protein
LA2395	847.3	1,736	COG2815S	putative outermembrane protein
LA2413	540.7	381.2	COG0791M	Probable lipoprotein nlpC precursor
LA2464	362	435.7	COG3225N	gliding motility protein GldG
LA2468	3,653	6,205	COG1196D	hypothetical protein
LA2510	1,230	846	COG1452M	hypothetical protein
LA2537	1,329	1,304	-	hypothetical protein
LA2538	624.2	606.5	-	hypothetical protein
LA2612	532.8	574	COG3190N	flagellar protein required for flagellar formation
LA2617	656.5	697.8	-	hypothetical protein
LA2656	1,128	637.2	COG2968S	hypothetical protein
LA2664	905.3	867.8	COG1706N	flagellar P-ring protein precursor
LA2672	662.3	1,116	-	hypothetical protein
LA2741	1,649	916.7	-	hypothetical protein
LA2742	814.8	524.2	-	hypothetical protein
LA2755	4,175	2,808	COG0768M	probable penicillin-binding protein
LA2757	1,213	1,270	COG1792M	rod shape-determining protein mreC
LA2800	665	1,591	-	hypothetical protein
LA2818	681.7	440.8	-	hypothetical protein
LA2857	506.7	516.5	COG0596R	Predicted hydrolase or acyltransferase, alpha/beta hydrolase superfamily
LA2949	407.5	460	COG0265O	heat shock protein, HtrA1
LA3069	1,221	786.3	-	hypothetical protein
LA3091	995.5	879.7	-	hypothetical protein
LA3118	771.7	1,239	COG0466O	hypothetical protein
LA3149	608.3	421.8	COG1629P	Hemin receptor
LA3165	749.2	454.7	COG4642S	conserved hypothetical protein with MORN repeat
LA3353	432.2	698.2	-	hypothetical protein
LA3403	391.3	388.8	-	hypothetical protein
LA3434	724.7	625.7	COG0860M	N-acetylmuramoyl-L-alanine amidase
LA3440	915.5	864.3	COG0237H	hypothetical protein
LA3468	618	584.8	COG1629P	probable TonB-dependent receptor
LA3469	658.2	685.7	COG3487P	iron-reglulated protein A
LA3506	1,407	1,266	COG0840NT	Methyl-accepting chemotaxis protein
LA3552	1,652	2,900	-	hypothetical protein
LA3632	1,028	1,337	COG1413C	PBS lyase HEAT-like repeat containing protein
LA3681	463.5	459	-	phage-related-like protein
LA3744	1,318	840.2	-	hypothetical protein
LA3862	537.3	761.3	COG0532J	hypothetical protein
LA3872	385.3	648.8	COG0616OU	Putative signal peptide peptidase sppA
LA3938	589	1,026	COG0457R	hypothetical protein
LA3970	532.7	398.5	-	hypothetical protein
LA4070	9,764	5,630	-	hypothetical protein
LA4212	1,678	1,684	-	hypothetical protein
LA4227	2,465	1,953	COG5621R	hypothetical protein
LA4232	509.3	612.2	COG2982M	hypothetical protein
LA4261	485.7	612.5	COG0451MG	UDP-glucose 4-epimerase
LA4263	1,012	1,290	-	hypothetical protein
LA4285	726.3	791.2	COG3858R	hypothetical protein
LA4341	1,009	1,146	COG0739M	Peptidase family M23/M37
LB018	1,549	1,589	COG1635H	hypothetical protein
LB025	371.8	382.5	-	hypothetical protein
LB050	344	533.3	-	hypothetical protein
LB056	443.5	523.8	COG0457R	TPR-repeat-containing protein
LB061	550.2	769.7	COG3211R	hypothetical protein
LB191	344.3	410	COG1629P COG4771P	putative TonB-dependent outer membrane receptor protein
LB199	917.3	925.2	COG1629P	putative outermembrane protein
LB258	552.5	1,082	COG4870O	Cysteine protease
LB277	1,634	984.3	-	hypothetical protein
LB279	1,115	804.3	COG1629P	hypothetical protein
LB328	1,591	2,672	COG1360N COG2885M	outer membrane protein OmpA
LB362	1,246	7,69	-	hypothetical protein

**Table 3:** The result of vaccine candidates according to localization sites: periplasmic

gene	Cy3 signal	Cy5 signal	COG	product
LA0430	2,614	2,094	COG1830G	hypothetical protein
LA0011	1472.2	2164	-	putative lipoprotein
LA0093	963.2	539.3	-	hypothetical protein
LA0107	476	466.3	-	hypothetical protein
LA0222	9,873	18,863	COG2885M	outer membrane protein OmpA family
LA0312	526.2	366.7	COG0739M	M23/M37 family protein
LA0413	505.3	544.2	-	hypothetical protein
LA0494	551	1,165	-	hypothetical protein
LA0569	404.2	366.3	-	hypothetical protein
LA0616	8,877	7,462	COG0457R	outer membrane lipoprotein lipL41
LA1118	610.2	614.3	-	putative outermembrane protein
LA1136	636.5	1,301	COG2834M	hypothetical protein
LA1155	534.3	563.8	COG1613P	sulfate-binding protein precursor
LA1312	1,514	1,070	-	hypothetical protein
LA1448	1,090	1,857	COG1464P	putative outermembrane protein
LA1998	676	700.8	COG0726G	polysaccharide deacetylase
LA2023	622	405	COG2010C	cytochrome c
LA2208	2,252	2,334	COG3858R	hypothetical protein
LA2277	609.5	391.3	-	hypothetical protein
LA2316	633.3	707.2	-	putative outermembrane protein
LA2372	1,427	2,257	COG2165NU	General secretory pathway protein G
LA2531	1,177	894.5	COG1196D	hypothetical protein
LA2637	51,707	37,602	-	LipL32 protein
LA2748	714.5	537.3	COG1613P	Sulfate-binding protein precursor
LA2820	691.3	525.5	-	hypothetical protein
LA2950	373.8	661	COG0265O	HtrA2
LA2993	349	433.8	-	hypothetical protein
LA3507	1,360	721.7	COG2010C	putative cytochrome c
LA3535	541.2	659.8	-	hypothetical protein
LA3571	607.2	492.8	COG2010C	putative cytochrome c
LA3576	595.8	594.5	COG1360N	flagellar motor protein
LA3780	1,365	1,432	-	hypothetical protein
LA3839	664	618.3	COG1881R	Phosphatidylethanolamine-binding family protein
LA3944	507.3	595.2	-	hypothetical protein
LA4262	355	515.8	-	hypothetical protein
LB047	506.3	2,137	COG2849S	hypothetical protein
LB098	735.5	507.3	COG0726G	Predicted xylanase/chitin deacetilase

**Table 4:** The result of vaccine candidates according to localization sites: innermembrane

gene	Cy3 signal	Cy5 signal	COG	product
LA0238	662.5	433.2	COG1612O	cytochrome-c oxidase assembly factor ctaA
LA0250	651.2	738.8	COG4956R	TRAM family protein
LA0314	577.2	368	COG0168P	Trk system potassium uptake protein trkH
LA0550	1,353	886.5	COG0841V	NolG efflux transporter
LA0639	858.2	469.7	-	hypothetical protein
LA0650	870.7	628	COG0705R	Rhomboid family protein
LA0680	530.2	707.5	COG0004P	hypothetical protein
LA0960	760.7	452	-	hypothetical protein
LA1056	702.7	607.8	COG0840NT	hypothetical protein
LA1143	4,027	4,074	COG0341U	Preprotein translocase subunit SecF
LA1191	1,014	790.7	COG0840NT	Methyl-accepting chemotaxis protein
LA1283	902.2	1,162	COG0845M	hypothetical protein
LA1284	415.7	543	COG4591M	Lipoprotein releasing system transmembrane protein lolC
LA1321	374.8	860.8	COG4232OC	thiol:disulfide interchange protein DsbD
LA1397	722.8	672.3	COG1033R	putative Protein export membrane protein SecD/SecF
LA1435	612.2	524.3	COG0392S	hypothetical protein
LA1451	415.2	435.2	COG1183I	Phosphatidylglycerophosphate synthase
LA1471	3,360	7,809	COG3808C	Pyrophosphate-energized vacuolar membrane proton pump
LA1477	566.7	436.8	COG1519M	3-deoxy-D-manno-octulosonic-acid transferase
LA1535	521.5	685.5	-	hypothetical protein
LA1554	498.7	398.2	COG1502I	hypothetical protein
LA1695	4,493	2,360	-	CrcB-like protein
LA1958	2,663	1,551	COG0526OC	putative outermembrane protein
LA1979	483.8	657.8	COG0463M	Putative glycosyl transferase
LA1982	342.5	452	COG3307M	hypothetical protein
LA2050	411.3	848.8	COG0707M	UDP-N-acetylglucosamine:LPS N-acetylglucosamine transferase
LA2250	10,742	9,624	-	Nuclease S1
LA2275	1,415	1,071	COG0586S	dedA protein
LA2320	1,319	1,496	COG0811U	biopolymer transport protein, putative
LA2604	464.3	448.7	-	hypothetical protein
LA2737	3,813	2,157	COG0204I	putative acyltransferase
LA2891	5,229	3,140	COG1055P	hypothetical protein
LA3072	1,970	1,665	COG0477GEPR	hypothetical protein
LA3110	1,262	2,371	COG2156P	potassium-transporting ATPase, C chain
LA3146	877	523.2	COG2076P	hypothetical protein
LA3577	1,618	1,198	COG1291N	motility protein A
LA3586	2,348	1,746	COG4270S	hypothetical protein
LA3754	667.3	449.7	COG0681U	Signal peptidase I
LA3777	497.3	539	COG0239D	Protein crcB homolog
LA3806	2,116	2,869	COG0004P	Probable ammonium transporter
LA3916	5,518	5,510	-	hypothetical protein
LA3926	967.8	1,802	COG0841V	transmembrane efflux pump protein
LA4062	1,326	2,138	-	hypothetical protein
LA4154	638.7	759	COG3225N	hypothetical protein
LA4155	1,140	1,015	COG1277R	probable permease of ABC transporter
LA4172	411	392.5	-	hypothetical protein
LA4228	559.5	627.8	COG4174R	Dipeptide transport system permease protein dppB
LA4233	409	985.7	COG1172G	hypothetical protein
LA4269	1,907	2,240	COG2207K COG0477GEPR	transcriptional regulator, AraC family
LB174	2,150	3,440	COG0501O	heat shock protein HtpX
LB281	5,026	2,708	COG0811U	transport protein ExbB

**Table 5:** Bacterial strains used in the study

serogroup	serovar	strain
Icterohaemorrhagiae	Lai	Lai(56601)
Canicola	Canicola	Lin
Pyrogenes	Pyrogenes	4
Autumnalis	Autumnalis	Lin 4
Australis	Australis	65-9
Pomona	Pomona	Luo
Grippotyphosa	Linhai	Lin 6
Hebdomadis	Hebdomadis	P 7
Bataviae	Paidjan	L 37
Sejroe	Wolffi	L 183
